# Precision Hemodialysis Using Sorbents to Remove Protein-Bound Uremic Toxins

**DOI:** 10.34067/KID.0000000000000168

**Published:** 2023-06-29

**Authors:** Stephen R. Ash

**Affiliations:** 1 Indiana University Health Arnett Inc., Nephrology, Lafayette, Indiana; 2 HemoCleanse Technologies LLC, Lafayette, Indiana

**Keywords:** dialysis, liver failure, uremia

In acute and severe kidney failure, hemodialysis is remarkably effective in improving symptoms of uremic coma, heart failure, fluid overload, hyperkalemia, and acidosis. However, long-term consequences of ESKD, such as inflammation, cardiovascular disease, mental deterioration, and asthenia, are not diminished and may even worsen with hemodialysis therapy. Protein-bound uremic toxins (PBUTs), unable to traverse the dialysis membranes as small toxins do, are believed to cause or contribute to these clinical manifestations. To supplement the functionality of contemporary hemodialysis machines, a few investigators have set off to design a device that affords the clearance of PBUT without interfering with the clearance of small and charged uremic toxins (SCUT).

In this issue of *Kidney360*, Meyer *et al.* show how to make a dialysis device highly selective for PBUTs.^1^ Surprisingly, their technique does not use formerly developed systems with a sorbent compound in suspension and powdered carbon outside the dialysis membrane as in BioLogic-DT (Liver Dialysis Unit)^2^ or fractionated plasma separation and adsorption as in Prometheus^3^ or a dialysate-containing albumin for sorption as in the molecular adsorbent recirculating system (MARS),^4^ or a hemofiltration membrane with a sorbent column to remove the toxins from filtrate as in the Supra-Hemofiltration with Reinfusion device.^5^ Rather, this simple but very effective PBUT-removing device comprises two large standard permeability dialyzers, a high dialysate flow of 1000 ml/min, and dialysate regeneration by a carbon block column (powdered carbon in a polymeric scaffold). Powdered charcoal strongly binds large, uncharged organic compounds, such as PUBTs and most middle molecular weight toxins (MMWT), without binding small uremic toxins such as urea.^6^

Patzer pioneered the idea that high dialysate flow through a standard high-permeability dialyzer could effectively remove protein-bound toxins and MMWT from blood. When MARS was first introduced to treat patients with hepatic failure and coma, it was believed that its effective removal of protein-bound toxins of liver failure was due to albumin, which somehow entered the membrane to directly contact plasma proteins and transfer toxins to the albumin. Doubting this idea, Patzer performed theoretical analyses to show that the removal of the PBUT was, in fact, but due to binding of the toxins by albumin or powdered carbon in the dialysate just outside the dialyzer membranes. This binding reduced the concentration of toxins in the dialysate to nearly zero and thereby improved the diffusion of toxins out of the blood. Patzer predicted that, for most PBUTs, a large dialysis membrane and high dialysate flow (such as 1000 ml/min) would accomplish the same clearances of protein-bound compounds as the MARS, Prometheus, and BioLogic-DT machines.^7,8^

Meyer and colleagues have proven that the prediction of Patzer was correct—in a study of PBUT removal rather than liver failure toxins.^1^ In his studies, a conventional system provided clearances of approximately 18 ml/min for p-cresol sulfate and 19 ml/min for indoxyl sulfate when dialyzing blood with these tightly bound solutes. The dialyzers with the carbon-block recirculating system had clearances of approximately 45 ml/min for p-cresol sulfate and 61 ml/min for indoxyl sulfate when operating alone, without removing small toxins such as urea. When operated in series, the clearances of the carbon-regenerated dialysis system and regular dialysis system had clearances for PBUT that were additive.^1^

Addition of an activated carbon block to the dialysis apparatus has implications for all types of dialyses as we now perform it. If a portion of effluent dialysate from any dialyzer were diverted through a carbon block column and then pumped into the inflowing dialysate, the dialysate use rate could remain the same, but the dialysate flow through the dialyzer would be increased. As a result, the clearance of PBUT and MMWT could be improved for any dialysis system, without need to increase dialysate usage.^9^ In dialysis systems with a dialysate usage of 300–400 ml/min, there would be a considerable increase in the clearance of PBUT and MMWT by increasing the dialysate flow rate through the dialyzer to 1000 ml/min and regenerating most of the dialysate with the carbon block. There would be a more dramatic increase in clearance if the dialysate usage were 30 ml/min, akin to settings used in continuous veno-venous hemodialysis (CVVHD). At dialysate flows of 30 ml/min, clearance for MMWT the size of vitamin B12 and *β* two microglobulin are approximately 10 ml/min; PBUT clearances are lower. Increasing dialysate flow to 250 ml/min followed by its complete regeneration would markedly increase PBUT clearance. This setting could be accomplished in CVVHD by running the effluent dialysate through a sterile carbon block on the way back to the 5-L dialysate bag. The bags of dialysate would be changed at a frequency required to maintain the removal of urea, phosphate, and potassium and to deliver bicarbonate as needed. This dialysis system would allow the physician to independently control the clearances of SCUT, MMWT, and PBUT. Eight hours of this type of therapy daily would result in better removal of PBUT and MMWT than 24 hours of standard CVVHD and provide adequate—not excessive—removal of SCUT, thus avoiding costly depletion and replacement of vital electrolytes. Carbon-block dialysate regeneration could make CVVHD better and less expensive for everyone involved, including patients, nurses, and doctors.^9^

What should be done from here? Clinical trials testing the PBUT module would greatly add to our understanding of the importance of PBUT in uremia. In the control group, a standard dialysis system would be operated unchanged. In the intervention group, the PBUT removal module would be added to the inflow blood line of the standard system. This PBUT module would include a very large dialyzer, a carbon block column for dialysate regeneration, and a pump circulating dialysate from the dialyzer through the carbon block and back to the dialyzer at 1000 ml/min. Such a comparative effectiveness clinical trial would test whether improved removal of PBUT resulted in better clinical outcomes.

As with any technology, benefit materializes only if new techniques are commercialized and made widely available. That is when inventions become innovations. There is a major barrier to innovations in dialysis because companies are invested in and market current technology, and they are thus reluctant to change the technology.^10^ However, all companies respond to a clamor from the market for improvements. Most physicians, nurses, and patients are far too complacent with the practice of dialysis as is performed now and too tolerant of the high cost, inconvenience, and poor outcomes. In the words of Walt Kelly through Pogo in 1971, “We have met the enemy and he is us.”
